# The Tip of an Iceberg: Replication-Associated Functions of the Tumor Suppressor p53

**DOI:** 10.3390/cancers10080250

**Published:** 2018-07-28

**Authors:** Vanesa Gottifredi, Lisa Wiesmüller

**Affiliations:** 1 Fundación Instituto Leloir, Consejo Nacional de Investigaciones Científicas y Técnicas. Av. Patricias Argentinas 435, 1405 Buenos Aires, Argentina; 2 Division of Gynecological Oncology, Department of Obstetrics and Gynecology of the University of Ulm, Prittwitzstrasse 43, 89075 Ulm, Germany

**Keywords:** POL iota, POL teta, RAD52, MRE11, ZRANB3, translesion DNA synthesis, template switching, fork reversal, mutant p53, therapy resistance

## Abstract

The tumor suppressor p53 is a transcriptional factor broadly mutated in cancer. Most inactivating and gain of function mutations disrupt the sequence-specific DNA binding domain, which activates target genes. This is perhaps the main reason why most research has focused on the relevance of such transcriptional activity for the prevention or elimination of cancer cells. Notwithstanding, transcriptional regulation may not be the only mechanism underlying its role in tumor suppression and therapeutic responses. In the past, a direct role of p53 in DNA repair transactions that include the regulation of homologous recombination has been suggested. More recently, the localization of p53 at replication forks has been demonstrated and the effect of p53 on nascent DNA elongation has been explored. While some data sets indicate that the regulation of ongoing replication forks by p53 may be mediated by p53 targets such as MDM2 (murine double minute 2) and polymerase (POL) eta other evidences demonstrate that p53 is capable of controlling DNA replication by directly interacting with the replisome and altering its composition. In addition to discussing such findings, this review will also analyze the impact that p53-mediated control of ongoing DNA replication has on treatment responses and tumor suppressor abilities of this important anti-oncogene.

## 1. Early Evidences of Transcription-Independent Roles of p53 in DNA Replication, Recombination and Repair

Approximately a decade after its discovery in 1979, p53 was shown to transcriptionally activate genes involved in cell cycle arrest and apoptosis upon various cellular stress situations [1]. Since then a wide range of p53 target genes has been described covering all thinkable biological functions ranging from metabolism to DNA repair [2,3]. Because oncogenic *TP53* mutations are closely associated with loss of transcription activation, a direct association between its role in transcription and its tumor suppressor ability was established. However, evidence obtained in mouse models deleting prime p53 target genes or expressing separation-of-function mutants of p53 with defective transcriptional activation but unaltered tumor suppression cast doubts on the exclusiveness of this canonical view (for review see [4]). Moreover, in the end of the 90s a number of reports provided evidence for additional activities of p53 in DNA replication, repair, and recombination, which could be separated from its transcriptional functions (reviewed in [5,6,7]). For instance, physical interactions of p53 with the base excision repair proteins POLß, Apurinic/apyrimidinic endonuclease (APE1), promote base excision repair (reviewed in a previous paper [6]). In parallel, several groups demonstrated a role of p53 in repressing aberrant homologous recombination repair of DNA double-strand breaks (DSBs) (reviewed in previous papers [5,6,7]), which required physical interactions of p53 with the human single-stranded DNA-binding protein RPA and the recombinase RAD51. Several lines of evidence supported the view that repression of homologous recombination could be separated from p53’s transcriptional activation. First, p53 mutant proteins which had lost transcriptional activation abilities, but partially retained homologous recombination repression (143V, 138V, and 22Q/23S) were identified. Second, overexpression of MDM2 (murine double minute 2) was shown to affect p53-dependent transcription but not p53-dependent repression of recombination. Third, the disruption of p53/RPA interaction by means of p53 mutations prevented its role in homologous recombination without affecting its ability to regulate transcription. Fourth, expression of a RAD51 mutant unable to bind p53 (186P), proved that p53’s antirecombinogenic function depends on the interaction with the recombinase [6,7]. Hence, a direct interaction of p53 with RAD51 has a negative effect on homologous recombination. 

In 1996, Mummenbrauer et al. discovered that the core domain of p53 harbors an intrinsic 3′-5′ exonuclease activity [8]. This finding, confirmed by other groups [9,10,11,12,13], was particularly intriguing because the majority of cancer-associated amino acid exchanges in p53 are localized in the core domain, certainly disrupting transcriptional activation by p53 but also having an effect on the exonuclease activity of p53. Dissection of the core domain led to the identification of a separation-of-function mutant protein p53(H115N) exerting transcription transactivation but no exonuclease activity [14]. Further in vitro data suggested auxiliary roles of p53 in the control of the fidelity of DNA synthesis by exonuclease-deficient polymerases and of homologous DNA exchange processes [11,13,15]. Subsequently, several pieces of evidence obtained in living cells brought forward the idea that p53 plays a role in replication-associated recombination events. Supporting such possibility, the analysis of p53 localization in cell nuclei revealed that p53 associates with DNA replication and recombination factors in response to replication stress [16,17]. At that time it was found that wild-type (wt) p53 stimulates homologous DNA exchange in mitotically growing cells under unperturbed conditions. Hence, p53 promotes homologous recombination during unperturbed replication but represses homologous recombination upon severe genotoxic stress that induces DSBs [18]. Further, siRNA screening performed in primary cells from *Trp53*-mutated mouse strains identified the contribution of both homologous repair and replication proteins to the homologous repair phenotype associated with breast cancer predisposition [19]. In summary, both in vitro and ex vivo data suggest an intricate relationship between the biochemical activities of p53 in DNA replication and recombination.

Meanwhile, a p53 role in other DNA replication-associated transactions was identified. Z. Livneh and coworkers demonstrated a role of p53 in the repression of translesion DNA synthesis (TLS) that is partially mediated by p21 but depends also on unknown functions of p53, possibly its exonuclease activity [20,21]. While such evidence suggested a direct role of p53 in DNA replication events, such p53 function remained enigmatic until recently. In the last few years, several groups had provided mechanistic evidences demonstrating how p53 affects DNA replication and replication-associated recombination processes. As will be discussed below, the core domain of p53 may also be required for the control of DNA replication by p53, having thereafter dual roles in DNA replication and transcription.

## 2. Current View: The Impact of p53 on DNA Replication

### 2.1. The Control of Nascent DNA Elongation by p53

While some manuscripts have lately explored a role of p53 in DNA replication, the use of the iPOND (isolation of proteins on nascent DNA technology) has recently demonstrated that p53 is yet another factor escorting ongoing replication forks [22]. This opens the possibility of a direct p53 role in the elongation of nascent DNA, a DNA replication parameter which is dysregulated by oncogenic stress and other genetic alterations [23]. 

**Figure 1:**
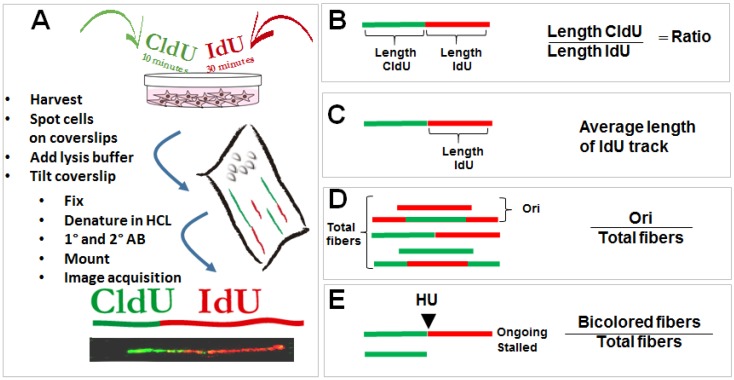
DNA spreading assay and protocols used to study the role of p53 in the regulation of DNA replication choreography. (**A**) Asynchronous cells are subjected to two subsequent incorporations of thymidine analogs (CldU and IdU). Immediately after the second pulse, samples are collected and harvested in denaturing conditions on a tilted coverslip. Cells are lysed while their content scrolls down the coverslip and DNA is spread on its surface. CldU and IdU are detected using specific antibodies. DNA track that incorporated the thymidine analogs are visualized as bicolored fibers. Detailed protocols can be found as described previously [25]. By determining the length of each track, a number of DNA replication parameters can be revealed. Examples that will be discussed in this review include (**B**) changes in the speed of the tracks which are usually associated with the accumulation of DNA lesions in the template DNA [26]; (**C**) the average speed for each track; (**D**) the frequency of origin firing defined as the frequency of origins fired during first and the second analog incorporation [27]; (**E**) the efficiency of replication restart, estimated by combining the second label with a replication blocking agent such as the inhibitor of ribonucleotide reductase, hydroxyurea. Different protocols were applied in the literature [28], but in the context of p53 a modified version of the restart protocol was used as detailed in this figure [22].

The study of DNA replication choreography has been prompted by the setup of DNA spreading and combing assays, a technology initially described previously [24] (see brief description of the assay in Figure 1A). Alternative versions of such protocol have been designed to evaluate different p53 transactions at the replisome (Figure 1B–E). 

**Figure 2:**
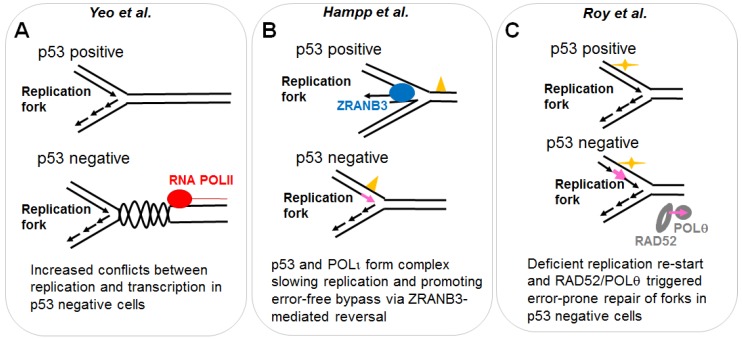
A potential direct role of p53 in DNA replication. The role of p53 in DNA replication was evaluated using the DNA spreading technology. (**A**) p53 prevents the generation and/or the stabilization of topological conflicts between transcription and DNA replication. The mechanism by which p53 prevents such conflicts is unknown. p53 deficient cells, which accumulate such type of conflicts on replicating DNA, rely heavily on Topo2. Inhibitors of such enzyme, selectively kill p53 deficient cells. (**B**) p53 positive cells are more prone to the activation of an error-free post-replication repair mechanism resulting in replication associated recombination, which likely depends on ZRANB3 mediated fork reversal [39]. Such an effect correlates with a reduction in nascent DNA elongation speed in p53 expressing cells and depends on the specialized DNA POLι, which interacts with p53. An H115N mutant of p53, impaired in its exonuclease activity, is incapable of interacting with POLι, promoting replication-associated recombination and restraining nascent DNA elongation. Yellow triangle: DNA damage caused by MMC. Pink arrow: Mutagenic DNA replication events, possibly dependent on specialized POLs. (**C**) p53 promotes DNA replication continuity and prevents the recruitment of RAD52 and POLθ. According to the literature RAD52 and POLθ should mediate error-prone repair of collapsed forks [49]. The use of H115N and S47P variants of p53 demonstrated that the control of DNA replication restart by p53 correlates with its tumor suppressor function independently of transcriptional activation. Yellow mark: HU treatment. Pink arrow: Mutagenic replication by POLθ and RAD52.

Using such an assay, three independent manuscripts have reported a potentially direct contribution of p53 (transcription independent) to the replication of DNA. Different cell lines and DNA damaging agents were used and results are not completely overlapping. Differences may result from the impact that p53 has on the choice of different DNA replication-auxiliary processes triggered by dissimilar types of DNA barriers on replication templates. By controlling such a choice, p53 improves the quality of DNA replication in terms of: (a) the selection of error-free pathways or (b) the coupling between DNA replication and other frequent DNA-associated transactions such as transcription (see Figure 2 and detailed explanation below). 

#### 2.1.1. p53 Favors the Coupling between Transcription and Replication Fork Progression

In 2016, Yeo et al. reported a cell death screening of 83 FDA (Food and Drug Administration) approved drugs that cause DNA damage in HCT116 colorectal cancer cells expressing or not expressing p53 [29]. It was found that p53 deficient cells were more sensitive than p53 proficient counterparts to Topoisomerase II (Topo2) inhibitors such as doxorubicin. Such sensitivity could be mediated in part by the inability of p53 deficient cells to arrest in G2/M [30]. However, it was also alleviated by the use of transcription inhibitors suggesting that p53 may regulate transcription-replication topological conflicts, possibly R-loops [31,32]. Moreover, accumulation of DSBs was observed in p53 deficient cells treated with Topo2-inhibitors, which were triggered by the accumulation of Topo2A-DNA complexes. Such a sensitivity of p53-deficient cells to replication-perturbing protein-DNA complexes is reminiscent of a previous study showing sensitivity of p53-deficient cells to poly (ADP-ribose) polymerase (PARP) inhibition [33]. Intriguingly, not only *Trp53*−/− cells but also MEFs from a p53(R172H) (corresponding to human p53(R175H)) mouse model [34] were sensitive to Topo2 and caused accumulation of Topo2 DNA complexes. Thus, a wtp53 function lost in the oncogenic variant seems to be necessary to mitigate Topo2-DNA complex formation. Transcriptional p53 targets such as p21 were shown not to be relevant for this novel function of p53 hence suggesting it may be independent from the transcription of at least this key p53 target. 

To evaluate if the loss of functional p53 increased the topological conflicts between transcription and DNA replication a DNA spreading assay was performed after incubation of cells with DRB (5,6-Dichloro-1-beta-Ribo-furanosyl Benzimidazole), a CDK7/9 inhibitor which blocks the elongation step of RNA-POLII transcription causing premature termination [35]. Two 15 min pulses of CldU (Chloro deoxyuridine) and IdU (Iodo deoxyuridine) were applied and bidirectional fibers analyzed. HCT116 *TP53*−/− cells displayed shorter labelled DNA tracks than the *TP53*+/+ counterparts suggesting that in the former cell line, replication forks were encountering replication barriers with increasing frequency. Strikingly, nascent DNA elongation increased in HCT116 *TP53*−/− cells treated with DRB reaching lengths similar to the ones observed in HCT116 *TP53*+/+. Hence, in *TP53*−/− cells, transcription inhibition facilitates DNA replication, suggesting that the replication barriers encountered by forks in p53 negative cells are transcription complexes or R-loops (Figure 2A). Consistently, persistent Topo2A complexes were also reverted by transcriptional inhibition in p53 deficient cells. The mechanism by which p53 coordinates DNA replication and transcription is yet unknown, but it is independent from at least one p53 target, the cyclin kinase inhibitor p21 [29].

#### 2.1.2. p53 Participates in the Choice of a Tolerance Pathway

A second report that indicates a role of p53 in DNA replication was published by us in the same year 2016 [36]. In that study Mitomycin C (MMC), a DNA damaging agent which causes intra and inter-strand cross-links on DNA, was used. Similar to PARP inhibitor treatment [33] or doxorubicin treatment [29], Hampp et al. found that p53 deficient cells were more sensitive to MMC than the p53 positive counterparts [36]. The protective effect of p53 upon MMC treatment was dissociated from the role of p53 as a transcriptional activator. In fact, in contrast to wt p53, the p53(H115N) mutant which is transcriptionally active but has an impaired exonuclease activity failed to prevent cell death. Also, various lines of evidence obtained both in tumor and primary cellular models (K562 leukemia cells, p53-mutated lymphoblastoid WTK1) showed that wtp53, but not the p53(H115N) mutant, promotes spontaneous DNA replication-associated recombination. Such a p53 effect, depends on POLι, a specialized DNA polymerase with low processivity and with a poorly characterized role in DNA replication [37]. p53 also forms a complex with POLι which is relevant for the elongation of nascent DNA [36]. Interestingly, DNA spreading assays (20 min CldU + 20 min IdU) demonstrated both p53 and POLι were required to constrain replication elongation both in unperturbed conditions and after treatment of cells with MMC. Such DNA spreading assays were performed in K562, H1299 lung carcinoma, U2OS osteosarcoma, and primary human CD34+ hematopoietic stem and progenitor cells after transient elimination or restoration of p53, whereby care was taken that expression levels did not exceed endogenous p53 levels [36]. Results are in apparent contradiction with the positive effect of p53 on nascent DNA elongation reported previously [29]. The different cell lines used in the two manuscripts and/or the potentially different adaptation of cells to transient [36] versus stable p53 loss [29] may be possible factors that cause such different outcomes. However, the type of DNA damaging agent used (Topo2 inhibitors versus MMC) might as well be critical. For instance, in their HCT116 cell system changes in the DNA damaging agent cause strikingly different effects on cell death, e.g., p53 protects cells from Topo2A inhibition and promotes cell death after MMC [29]. Conversely, H1299 cells expressing inducible p53 were more resistant to PARP inhibitor and MMC but displayed higher sensitivity to DSB-inducing ionizing radiation [33,36]. Since the drug desensitizing effects of p53 correlate with effects of p53 on nascent DNA elongation it is tempting to speculate on a causal relationship between fork elongation and cell survival.

Mechanistic evidences reported by Hampp et al. [36] indicate that the p53/POLι complex may escort the replication fork creating idling cycles [38]. Such cycles involve a DNA synthesis step (in charge of POLι) and a DNA degradation step (in charge of the p53 exonuclease activity) which may prolong the time-window for the pathway choice for DNA damage tolerance. Most interestingly, it was demonstrated that the p53/POLι complex favors a type of post-replication repair involving HLTF (helicase like transcription factor), a Rad5-ortholog known to mediate PCNA (proliferating cell nuclear antigen) polyubiquitination, and ZRANB3 (zinc finger RANBP2_Type containing 3), a translocase which more recently was demonstrated to cause replication fork slowing and reversal upon PCNA polyubiquitination [39]. If replication stalling is persistent, p53 but not p53 (H115N) may be capable of promoting a more extended remodeling of stalled forks by the exonuclease activity of MRE11 [36] in a manner that is reminiscent of previously described dysregulated exonuclease activity of MRE11 at forks [40]. Hence, in a manner that depends on the amino acid critical for its exonuclease activity [14], p53 regulates the choice of pathways that ensure DNA replication continuity (Figure 2B) [36]. 

#### 2.1.3. p53 Promotes Replication Re-Start Preventing Mutagenic RAD52-Mediated DNA Repair

A third manuscript addressing a role of p53 at replication forks was published in 2018. Using iPOND technology, Roy et al. demonstrated that p53 escorts the replisome [22]. Such observation is in line with the proposal of a direct role of p53 at replication forks [36]. While this manuscript also utilizes the DNA spreading technology, the approach was slightly different and difficult to compare with the two previous manuscripts. In this case hydroxyurea (HU), a ribonucleotide reductase inhibitor which causes a depletion of dNTPs thereafter reducing the speed of DNA replication [41], was used. The authors took advantage of the rapid stalling of replication forks caused by HU treatment. Replication stalling was evaluated during HU treatment in HAP-1 fibroblast, H1299 tumor cells, and mouse embryo fibroblasts (MEFs) indicating that p53 facilitates replication continuity (interpreted as restart by the authors) and origin firing [22]. Use of two different oncogenic p53 mutants and a breast cancer predisposing variant allowed to establish that such a novel role of p53 is transcription independent. The authors concluded that a defect in a replication protection mechanism (the ability of forks to restart) correlated with tumorigenicity of p53 variants in humans. Such a result is puzzling given that tumorigenicity could be expected to be associated with advantages, and not with defects, in the ability of cells to adapt DNA replication to stress conditions. An elaborated analysis involving the use of SIRF (in situ interactions at replication forks using PLA) technology [42] revealed that p53 promotes the recruitment to stalled forks of the MLL3 chromatin remodeler and the MRE11 nuclease (which is in line with a previous study [36]). Such an alteration in replisome composition prevented the localization of RAD52 and POLθ to such EdU positive sites on the DNA [22]. The utilization of an inhibitor of MRE11 in SIRF suggested that p53 and MRE11 limit the mutagenic repair of replication forks by RAD52/POLθ (Figure 2C) [22]. In agreement with previous studies [29,36], this report also suggested that p53 may help to overcome impediments of nascent DNA synthesis. This could happen in a manner that is related to a role of p53 in the selection of the activities that escort the replisome. As suggested by Hampp et al. and Roy et al., p53 likely promotes remodeling at stalled forks facilitating replication continuity driven by nonmutagenic pathways [22,36].

### 2.2. Biological Relevance of the Participation of p53 in the Replisome

As discussed previously, p53 mitigates cell death after MMC treatment in H1299 cells [36] and after Topo2A inhibition in HCT116 cells [29], despite opposite effects on DNA replication speed when using MMC [36] and DRB [29]. However, DNA elongation still needs to be evaluated after Topo2A inhibition before precipitating conclusions. Given that MRE11 is mutated in HCT116 cells [43,44] and that such a nuclease is central for the models proposed previously [22,36], it is difficult to interpret those findings in simple side by side comparison with a previous study [29]. But, is replication speed indeed causal to drug sensitivities or those variables are just correlating? According to a previous study [29], ongoing forks in p53 deficient cells are overloaded with Topo2 because of an increase in the topological conflict between transcription and replication. Therefore, when compared to p53 proficient counterparts, impaired fork progression in p53 deficient cells treated with Topo2 inhibitors could be expected. An important evidence that supports a causal link between the effect of p53 on DNA replication and cell survival is that DRB not only elongates replication tracks in p53 deficient cells but also alleviates cell death induced by Topo2 inhibitors in p53 deficient cells. When focusing on other DNA damaging agents, the MMC results suggest that bulky adducts may pose different replication challenges to p53 positive and negative cells [29,36]. In this scenario, p53 reduces DNA elongation speed but, counterintuitively again, alleviates cell death after MMC [36]. However, p53 also promotes post-replication repair resulting in replication-associated recombination after MMC [36]. More work will be required to determine the relevance of the regulation of nascent DNA elongation rates by p53 for its tumor suppressor activity and drug resistance mechanisms in tumor cells. 

The experiments performed previously [22] were provocative in terms of the biological relevance of DNA replication restart by p53. First, they used different p53 variants that either retain or do not retain the transcriptional activity of p53 with the aim of exploring the correlation between transcriptional activation or DNA replication re-start with the tumor suppressor activity of p53. Intriguingly, a correlation between the compromised tumor suppressor activity of p53 and the DNA replication restart function of p53 was found. One of the p53 variants used in this study was p53(P47S), encoded by a polymorphism found in Africa, which modifies breast cancer susceptibility [45,46]. p53(P47S) revealed a separation-of-function since it is transcriptionally active and proficient for apoptosis [45,47], but it is deficient in the replication restart function of p53 [22]. Importantly, previous work has shown that residue 47 is situated in the RPA binding site, next to aa 48 and 49 mutated in a p53 separation-of-function mutant still active in transcription but no longer in repression of DSB-induced HR (homologous recombination) [48]. Notably, the p53(P47S) mutant was highly sensitive to HU and MMC and accumulated micronuclei in cells that transited S phase upon such genotoxic treatment, hence revealing the biological relevance of the replication restart by p53 [22].

Also, transcriptional independent functions of p53 may be relevant for the protection of cells against MMC [36]. The p53(H115N) mutant, a separation-of-function mutant unable to promote post-replication repair in complex with POLι but retaining transcription function did not mediate resistance to MMC like wtp53 [36]. In fact we speculate that findings by Hampp et al. and Roy et al. may be linked. In p53 proficient cells, p53-mediated post-replication repair by ZRANB3 and HLTF should prevent persistent replication stalling and possibly fork collapse [36]. In contrast, in p53 deficient cells, collapsed forks (which may increase as a consequence of p53 loss) may be subjected to dysregulated RAD52/POLθ usage [22]. When analyzing COSMIC (catalog of somatic mutations in cancer) mutational signatures comparing p53 positive and negative samples in the TCGA data base, one signature of >3 bp deletions with microhomologies at break junctions was compatible with RAD52 and POLθ mutation spectra. Therefore, the replication restart activity of p53 could play a role in the prevention of human breast cancer onset [22]. 

### 2.3. The Control of Nascent DNA Elongation by p53 Targets

**Figure 3:**
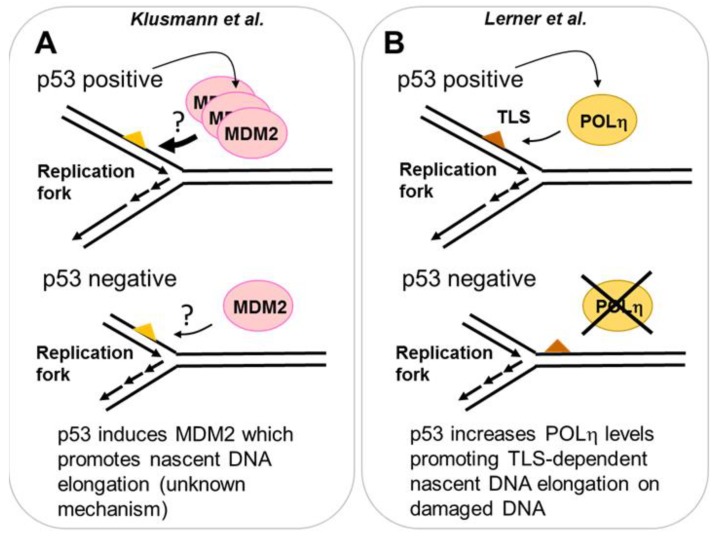
Transcription dependent role of p53 in DNA replication. (**A**) Using Nutlin-3a as an inducer of p53, a novel role of increased p53 levels on DNA replication was found. When Nutlin-3a treatment was combined with a source of replication stress (HU or gemcitabine), a role of MDM2 in the elongation of nascent DNA was revealed. Strikingly, in p53 negative cells, the MDM2 pathway that controls DNA replication is still active, despite the lack of p53-dependent induction of MDM2. The elimination of MDM2 in p53 negative cells reduces DNA elongation in such genetic background. (**B**) p53 induces the specialized POLη after UV irradiation. Cells with increased levels of POLη (generated by a pre-irradiation with low dose of UV) promote increased nascent DNA elongation. In p53 deficient cells, the p53 dependent induction of POLη is lost, nascent DNA elongation post-UV is impaired and the cell viability is reduced.

p53 can induce hundreds of targets that promote cell cycle arrest and cell death and many other outcomes relevant for its antioncogenic function [50]. It is therefore valid to examine whether the contribution of p53 to DNA replication can also be mediated by p53 targets. More than a decade ago, a role of the p53 target, the cyclin kinase inhibitor p21 in the regulation of the ubiquitination of PCNA was reported [21,51]. Such a post-transcriptional modification of PCNA promotes DNA damage tolerance events across DNA damage [52]. In particular, damage tolerance by translesion DNA synthesis promotes the loading of specialized DNA polymerases to the replisome. Such DNA polymerases aid DNA synthesis across damaged DNA, which are poor templates for replicative DNA polymerases [26]. In addition to the cyclin kinase binding domain, p21 has a strong PCNA binding domain that is critical for displacing specialized DNA polymerases from PCNA [53]. When DNA templates are damaged by UV irradiation, p21 must be removed from replication forks to promote their TLS-dependent progression [54]. Hence, on damaged DNA templates, p21 is a negative regulator of nascent DNA elongation. However, under unperturbed replication conditions, p21 is required at active forks to promote nascent DNA elongation. In that case, p21 prevents the dysregulated loading of specialized POLκ to undamaged DNA. If POLκ is recruited to undamaged templates it reduces replication speed as a consequence of its low processivity [55]. Hence, on undamaged DNA, p21 protects DNA replication elongation, preventing inefficient elongation by specialized DNA polymerases. It should also be mentioned that after the initial submission of this review a manuscript indicating that p21 also prevents nascent DNA elongation in cells treated with yet another source of DNA damage, PARP inhibitors, was published [56], an observation that is in agreement with a previous study [54]. However, in contrast to another previous study [55], the authors found a negative effect of p21 on the elongation of undamaged DNA [56]. While more work will be required to determine the nature of such contradictory results, it is clear that p21 is a key regulator of replication speed. Furthermore, the regulation of DNA replication by p21 is not limited to a direct control of the replisome. p21 overexpression (in tumor cells expressing mutant p53) alters origin firing and increases replication stress in a manner that depends on the reduced availability of replisome regulators such as the CRL4-CDT2 ubiquitin ligase [57]. Hence p53 and p21 are important regulators of the DNA replication choreography. In addition to p21 other two p53 targets, MDM2 and the specialized POLη, also contribute to DNA replication (see Figure 3 and detailed explanation below).

#### 2.3.1. MDM2 Is a p53 Target That Promotes Nascent DNA Elongation

MDM2 is the E3 ligase that triggers p53 degradation and is one of the most studied p53 targets [58]. Nutlin-3a is an MDM2 inhibitor thereby promoting p53 stabilization [59]. In contrast to genotoxins discussed before, Nutlin-3a does not directly induce DNA damage. However, Klusmann et al. used it to explore if p53 could exert a genome-protective function in S phase [60]. Prolonged incubation (14 h) of cells with Nutlin-3a caused the expected p53 stabilization and increased nascent DNA elongation. DNA fiber assays were mostly performed by incorporating both analogs after 14 h of Nutlin-3A treatment and using long labelling periods (20–60 min CldU—1–2 h of alternating IdU/CldU labelling in U2OS and HCT116 cancer cells, as well as in MEFs and primary murine thymocytes) [60]. Such a contribution of p53 to nascent DNA elongation was also observed when using inhibitors of ribonucleotide reductase such as gemcitabine and HU (in this case also shorter protocols-20 min CldU—20 min IdU were used in U2OS and primary thymocytes, revealing more modest differences in fork rate between p53 proficient and deficient samples when compared to long protocols). As 14 h of Nutlin-3A was necessary to modify the rate of DNA replication, the authors speculated that the regulator of such a DNA replication parameter was a p53 target [60]. Supporting this notion, MDM2 downregulation in p53 null cells contributed to nascent DNA elongation similarly to p53 inhibition (Figure 3A) [60]. As MDM2 downmodulation experiments were performed in p53 negative cellular models (HCT116 and MEFs) only we hypothesize that: (a) MDM2 must be a powerful regulator of DNA replication as low, p53 independent levels of MDM2 suffice to modulate DNA elongation rate, (b) p53 and MDM2 are not fully epistatic with respect to the regulation of DNA replication. Hence, the participation of other p53 domains and/or targets in the regulation of ongoing replication forks must be considered. 

#### 2.3.2. POLη Is a Target of p53 That Promotes Nascent DNA Elongation

The most common DNA lesion caused by UV irradiation are dimers between adjacent thymidines called cyclobutane pyrimidine dimers (CPDs) [61]. Replicative DNA polymerases cannot replicate across those DNA lesions but the specialized POLη can, and it does so with remarkable accuracy [62]. In humans, the loss of translesion DNA synthesis across CPDs by POLη causes a cancer predisposition syndrome called Xeroderma Pigmentosum Variant [63]. Interestingly, some years ago it has been reported that POLη is a p53 target [64] and a recent report by Lerner et al. demonstrated that POLη accumulation by p53 has a protective role in cells treated with UV irradiation [65]. Experiments performed using split doses of UV (pre-irradiation with low UV dose, followed by a lethal dose of UV delivered 24 h later) demonstrated that both p53 and POLη are induced by the first dose and are required to protect cells from cell death after the second dose of UV irradiation. Such an increase in cell survival correlated with the improved elongation of nascent DNA in cells treated with split-doses when compared with a single pulse of the high dose. Strikingly, the protective effect was lost when POLη or p53 were depleted (Figure 3B) [65]. Hence, p53-dependent upregulation of POLη after UV irradiation promotes nascent DNA elongation and cell survival. 

### 2.4. Biological Relevance of the Contribution of p53 Targets to DNA Elongation

Given the well characterized role of POLη in DNA replication across CPDs, the evaluation of the relevance of p53-dependent upregulation of POLη for DNA replication is rather simple. POLη may be a limiting factor in cells as its p53-dependent increase promotes cell survival [65]. It is unclear whether the p53-POLη pathway could be protective against other types of DNA damage. However, a contribution of POLη to post-replication repair mediated by ZRANB3 in p53 proficient cells has been excluded [36].

The mechanistic link between MDM2 and DNA replication is much more difficult to understand. It is not known whether MDM2 escorts the replication fork or if alternatively, modulates DNA replication indirectly. The activity/domain of MDM2 required for the control of nascent DNA elongation is also unknown. It is remarkable that the residual MDM2 levels in p53 deficient cells are sufficient to promote DNA elongation as knockout or knockdown of MDM2 in p53 deficient cells reduces nascent DNA elongation [60]. It could be hence predicted that subtle changes in MDM2 levels in replicating cells may have an impact on the DNA elongation rate and henceforward be extremely important for cell survival.

## 3. Concluding Remarks

**Figure 4:**
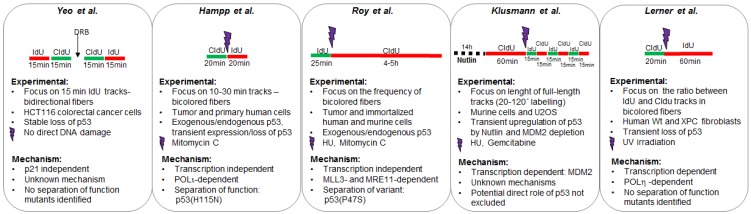
Different protocols used to explore the effect of p53 on DNA replication. The experimental design of each study is illustrated in the upper part of each panel. Other experimental settings are detailed in the mid-section. Mechanistic insights collected in each study are detailed in the bottom part of each panel.

This report focuses on roles of p53 that are directly linked to the control of DNA replication. However, p53 can also control the decision of S phase entrance. DDR (DNA damage response) induced p53 can be transmitted from mother to daughter cells influencing the proliferation-quiescence decisions of the latter [66]. Hence, by controlling the length of G1 phase and the timing of S-phase entry p53 may also affect DNA replication fidelity. However, the reports discussed in this review focus on a rather different contribution of p53 to DNA replication regulation, e.g., the ability of p53 to exert an intra S-phase control of DNA replication which may have tumor suppressor potential (see summary in Figure 4). Those reports also suggest that p53 may have more than one contribution to DNA replication, which could depend on different p53 domains and may include but may not be limited to the transcriptional activation of p53 targets genes. It is unclear if different DNA damaging agents may promote different combinations of DNA replication functions of p53, which in turn may trigger opposite biologically relevant outcomes. It is henceforth a simplification to linearly compare the results in different reports that used different fiber spreading protocols and diverse DNA damaging agents (or even agents that impose no apparent DNA damage). In fact, it should be mentioned that using 20 or 60 min of incorporation for the second analog was reported to completely invert the effect of RAD51 or FANCD2 depletion on the average length of the nascent DNA [67,68]. It must also be considered that the depletion of p53 could cause similar time-dependent alterations in the results. Another fact to consider is that the modulation of p53 levels is strictly associated with the modulation of the levels of its targets genes. Many of those proteins have also the power to control DNA elongation (p21, MDM2, POLη, and possibly others). Hence, slight changes caused perhaps by the type and prevalence of replication barriers may differentially affect the levels/localization of some of those proteins triggering DNA elongation results that may seem contradictory at first glance. Of note, replication-regulatory functions of wtp53 were unequivocally found to protect cells from DNA damage independently of the exact DNA elongation phenotype mediated by p53 [22,33,36,60,65]. These findings imply that p53 actions in DNA replication can also mediate resistance to cancer treatments, while others activate the canonical killer functions via transcription and apoptosis induction. Therefore, unraveling functions of p53 in DNA replication versus checkpoint control may indeed help to solve the riddle of p53 acting as a healer and a killer [33,69,70,71]. Much more work will be required to establish the role of p53 in DNA replication and the relevance of such a novel function of p53 in tumor suppression and treatment responses. The identification of separation of function mutants such as the ones described in [22,36] will be key to identify the molecular mechanisms that prompt p53-dependent control of DNA replication. A systematic analysis of p53 domains will be also required to establish if other domains of p53 (in addition to the exonuclease domain within the core) are required to regulate DNA replication. The direct effect of p53 mutants on DNA replication will also require further exploration as GOF (gain of function) mutants were already proven to alter the DNA replication choreography and the expression of replisome components [72,73,74]. It is also intriguing that the mutation of p53 has been recently associated with chromothripsis [75], a type of genomic instability that results from an inordinate number of translocations in one chromosome. The mechanism leading to chromothripsis is poorly understood but it is supposed to be generated in one single replication cycle, and it is expected to require the coordinated failure of a number of replication and repair pathways [76]. While a direct role of p53 in the prevention of catastrophic chromosomal breakage and rearrangement cannot be excluded, a p53 target gene, PTCH53, has also been involved in the control of the dysregulated hegedhog signaling pathway, which may be crucial to promote chromothripsis [77]. In summary, the manuscripts reporting a role of p53 in DNA replication describe the tip of an iceberg that we were not aware of. Intense research will be required to identify upstream players and interactions between p53 and its targets at the fork. Given the central role that DNA replication has on tumorigenesis, it will be relevant to establish whether the DNA replication function of p53 is also a tumor suppressor function of this important anti-oncogene.
